# Supplementary figures and images for: Epidemiological correlates of overweight and obesity in the Northern Cape Province, South Africa
Source: PeerJ. 2023 Feb 9;11:e14723. doi: 10.7717/peerj.14723 (PMC9922494; doi:10.7717/peerj.14723)

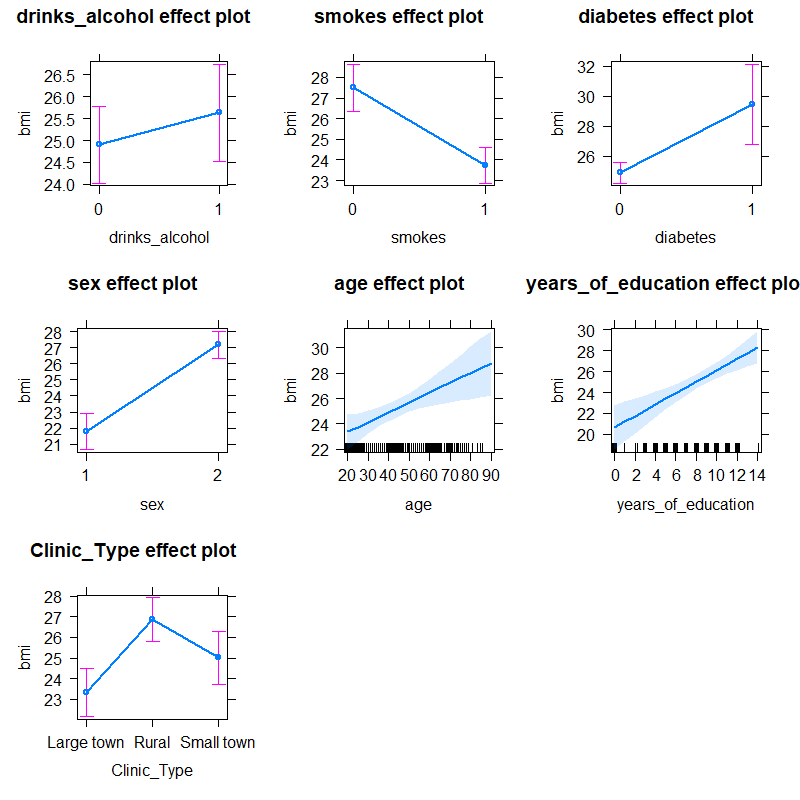

Supplement: Supplemental Information 1 [file peerj-11-14723-s001.png]
