# Supplementary material for: Epidemiological correlates of overweight and obesity in the Northern Cape Province, South Africa
Source: PeerJ. 2023 Feb 9;11:e14723. doi: 10.7717/peerj.14723 (PMC9922494; doi:10.7717/peerj.14723)
Supplement: Supplemental Information 2 [file peerj-11-14723-s002.docx]

| **Ethnicity** | **Count** |
| --- | --- |
| Coloured | 343 |
| Nama | 15 |
| Tswana | 12 |
| San | 5 |
| Nama/Damara | 3 |
| Damara | 3 |
| Xhosa | 3 |
| White | 1 |
| Other | 7 |

***Table S1:*** *Self-reported ethnicity data of participants enrolled in the present study.*
